# Supplementary material for: Quercetin modulates mast cell activation and the IL-33/ST2/NF-κB axis in allergic rhinitis
Source: Sci Rep. 2026 Jun 14;16:27159. doi: 10.1038/s41598-026-57981-6 (PMC13526780; doi:10.1038/s41598-026-57981-6)
Supplement: Supplementary file 1 [file 41598_2026_57981_MOESM1_ESM.pdf]

Supplementary Figure 1

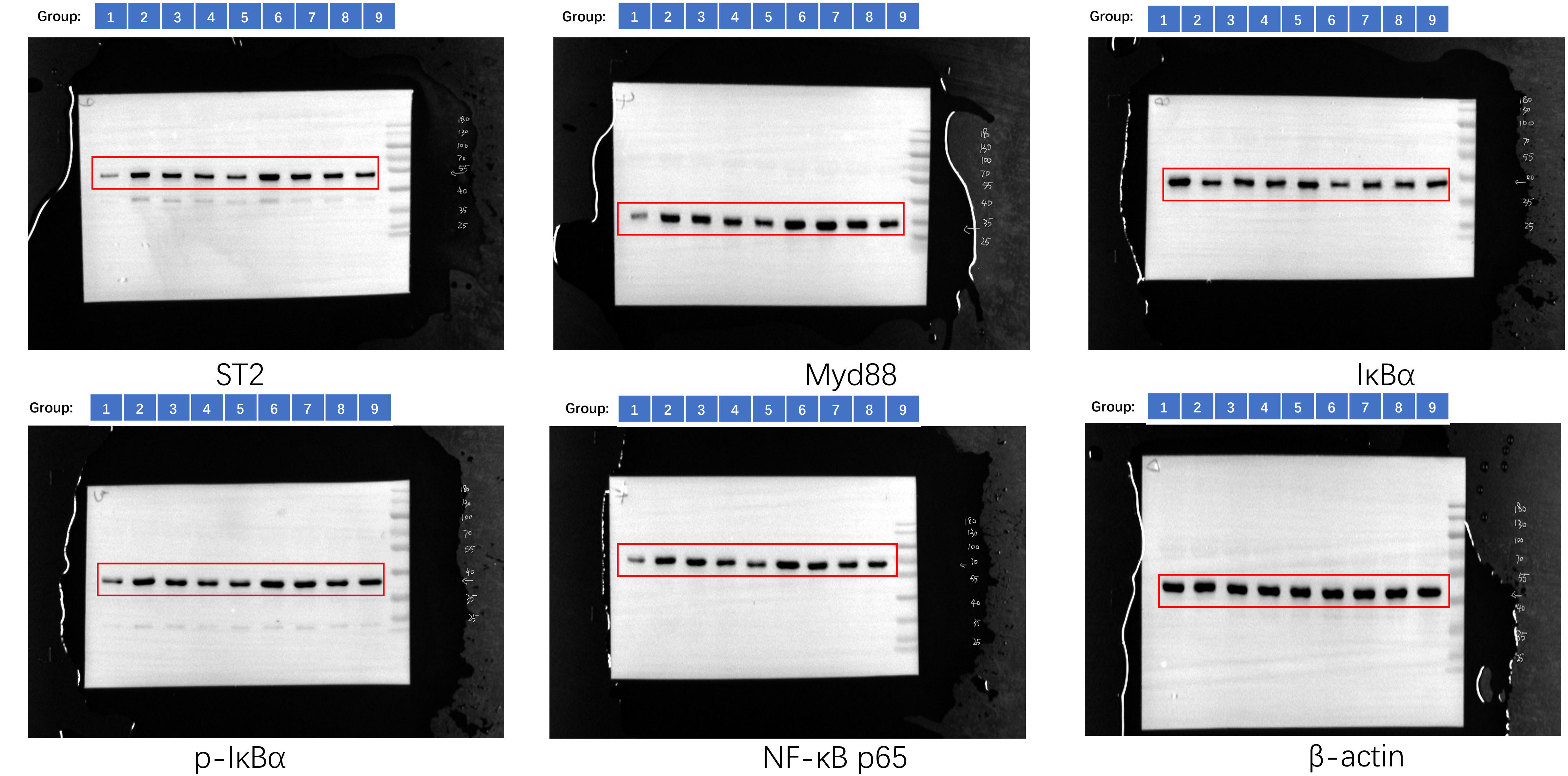

Raw_WB_Images For Fig2E：

Group 1: RBL-2H3 cells (untreated control).**Group 2:** RBL-2H3 cells + anti-DNP-IgE (500 ng/ml) + DNP-HSA (500 ng/ml).**Group 3:** RBL-2H3 cells + anti-DNP-IgE (500 ng/ml) + quercetin (10 μM) + DNP-HSA (500 ng/ml).**Group 4:** RBL-2H3 cells + anti-DNP-IgE (500 ng/ml) + quercetin (5 μM) + DNP-HSA (500 ng/ml).**Group 5:** RBL-2H3 cells + anti-DNP-IgE (500 ng/ml) + quercetin (1 μM) + DNP-HSA (500 ng/ml).**Group 6:** RBL-2H3 cells + anti-DNP-IgE (500 ng/ml) + DNP-HSA (500 ng/ml) + IL-33 (50 ng/ml).**Group 7:** RBL-2H3 cells + anti-DNP-IgE (500 ng/ml) + quercetin (10 μM) + DNP-HSA (500 ng/ml) + IL-33 (50 ng/ml).**Group 8:** RBL-2H3 cells + anti-DNP-IgE (500 ng/ml) + quercetin (5 μM) + DNP-HSA (500 ng/ml) + IL-33 (50 ng/ml).**Group 9:** RBL-2H3 cells + anti-DNP-IgE (500 ng/ml) + quercetin (1 μM) + DNP-HSA (500 ng/ml) + IL-33 (50 ng/ml)

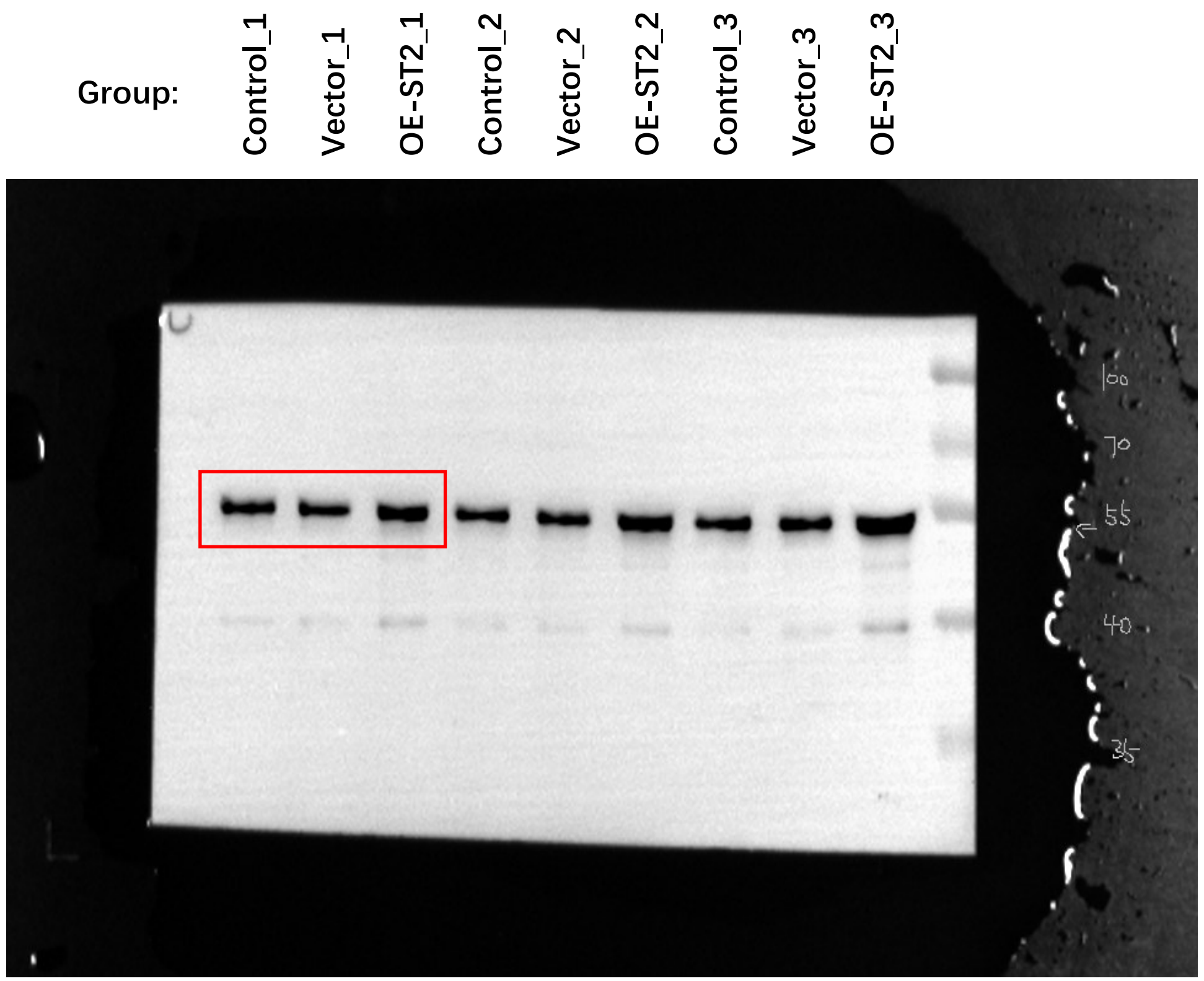

ST2

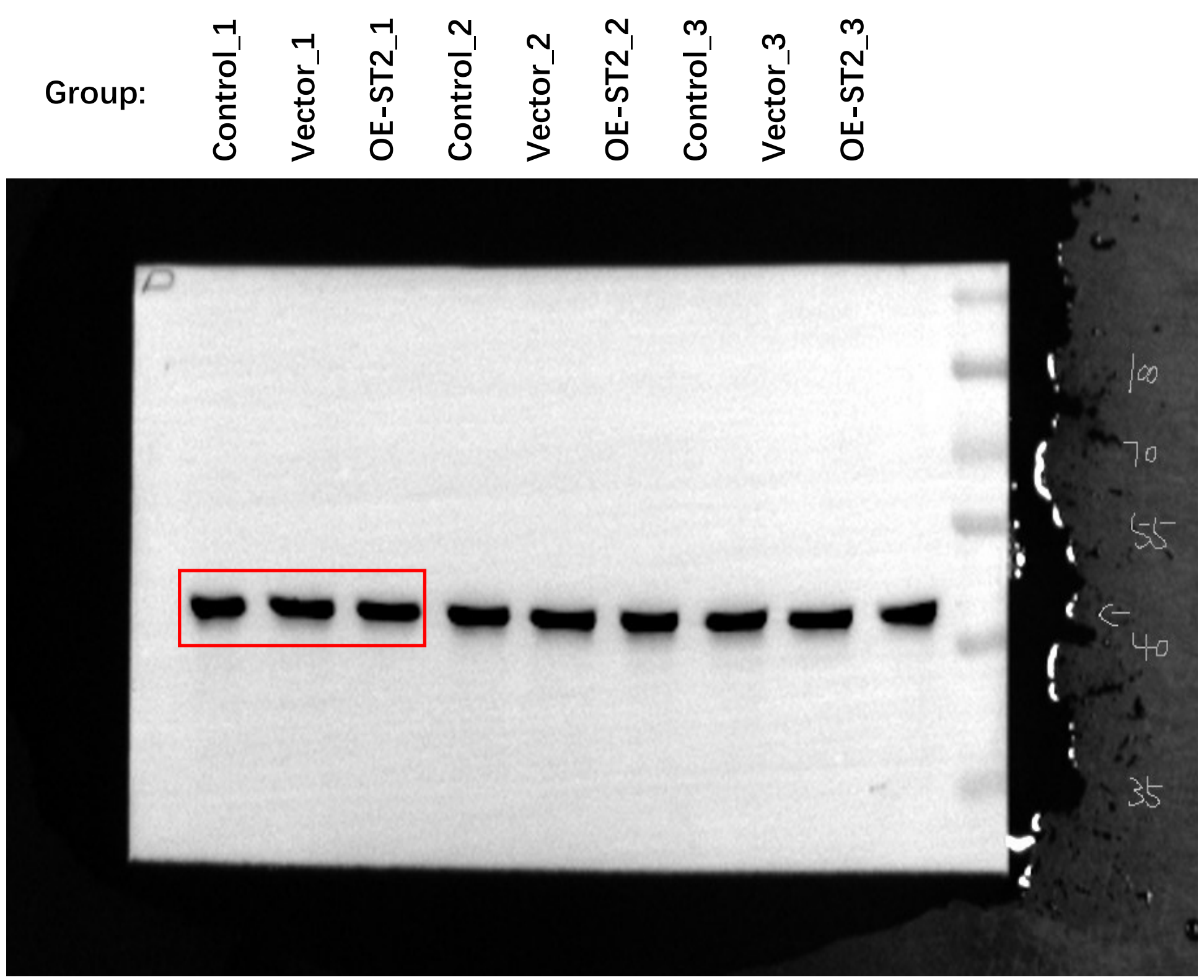

β-actin

Raw_WB_Images_Fig 3A

OE-ST2 :overexpression-ST2

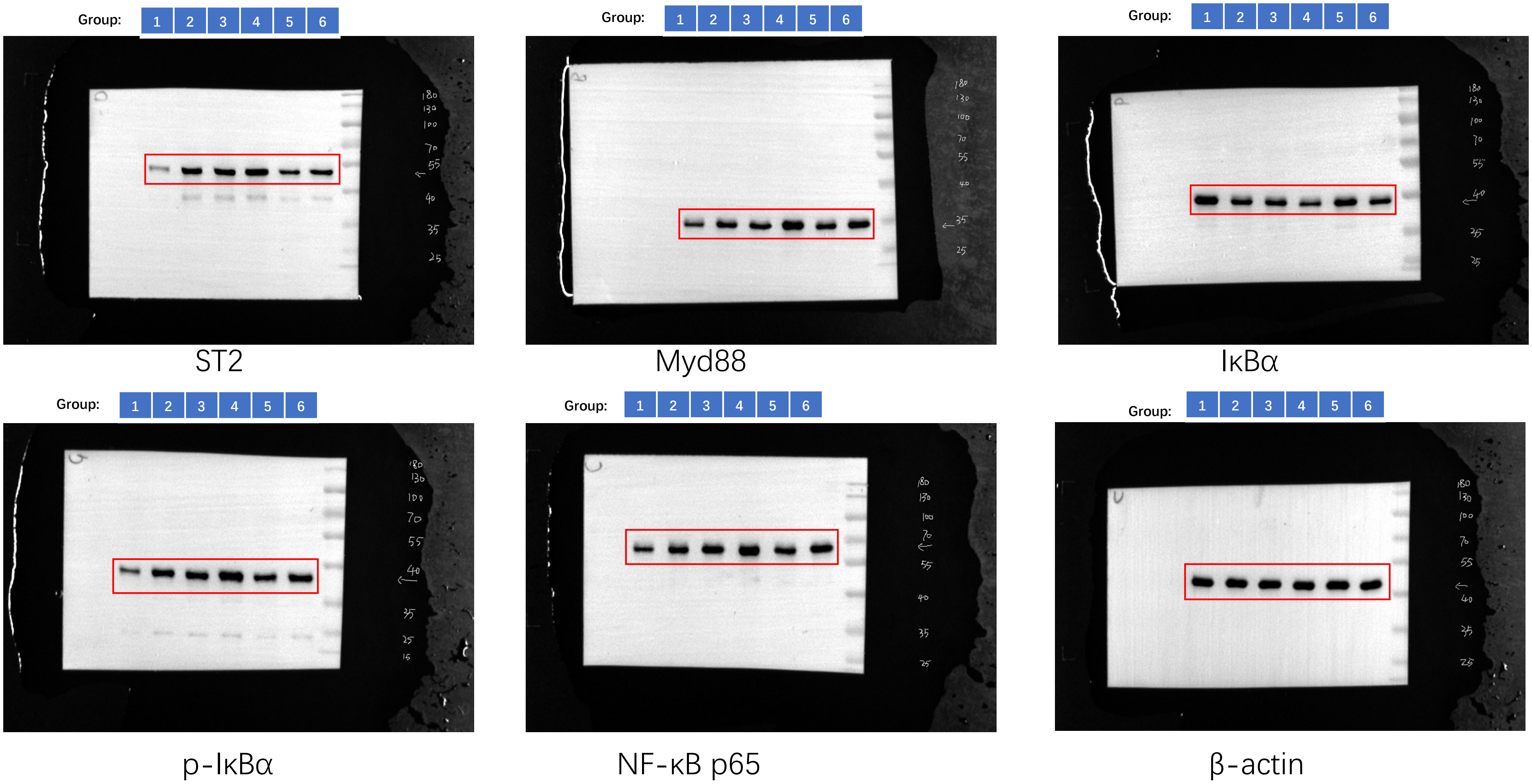

Raw_WB_Images_Fig3H

Group 1: RBL-2H3 cells only (untreated control).**Group 2:** RBL-2H3 cells + anti-DNP-IgE (500 ng/ml) + DNP-HSA (500 ng/ml) + IL-33 (50 ng/ml).**Group 3:** RBL-2H3 cells + overexpression-NC + anti-DNP-IgE (500 ng/ml) + DNP-HSA (500 ng/ml) + IL-33 (50 ng/ml).**Group 4:** RBL-2H3 cells + overexpression-ST2 + anti-DNP-IgE (500 ng/ml) + DNP-HSA (500 ng/ml) + IL-33 (50 ng/ml).**Group 5:** RBL-2H3 cells + overexpression-NC + anti-DNP-IgE (500 ng/ml) + DNP-HSA (500 ng/ml) + IL-33 (50 ng/ml) + quercetin (5 μM).**Group 6:** RBL-2H3 cells + overexpression-ST2 + anti-DNP-IgE (500 ng/ml) + DNP-HSA (500 ng/ml) + IL-33 (50 ng/ml) + quercetin (5 μM)

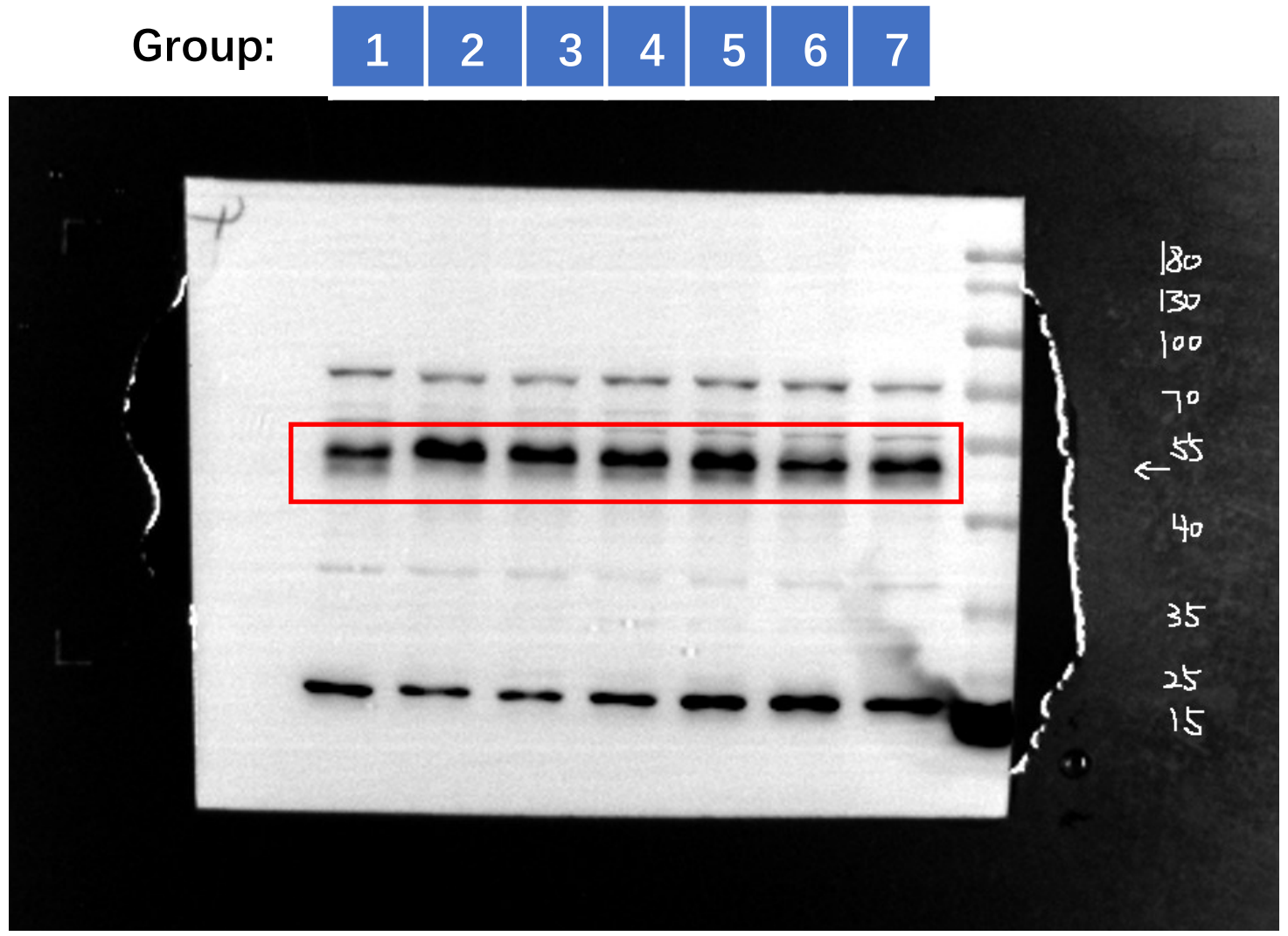

ST2

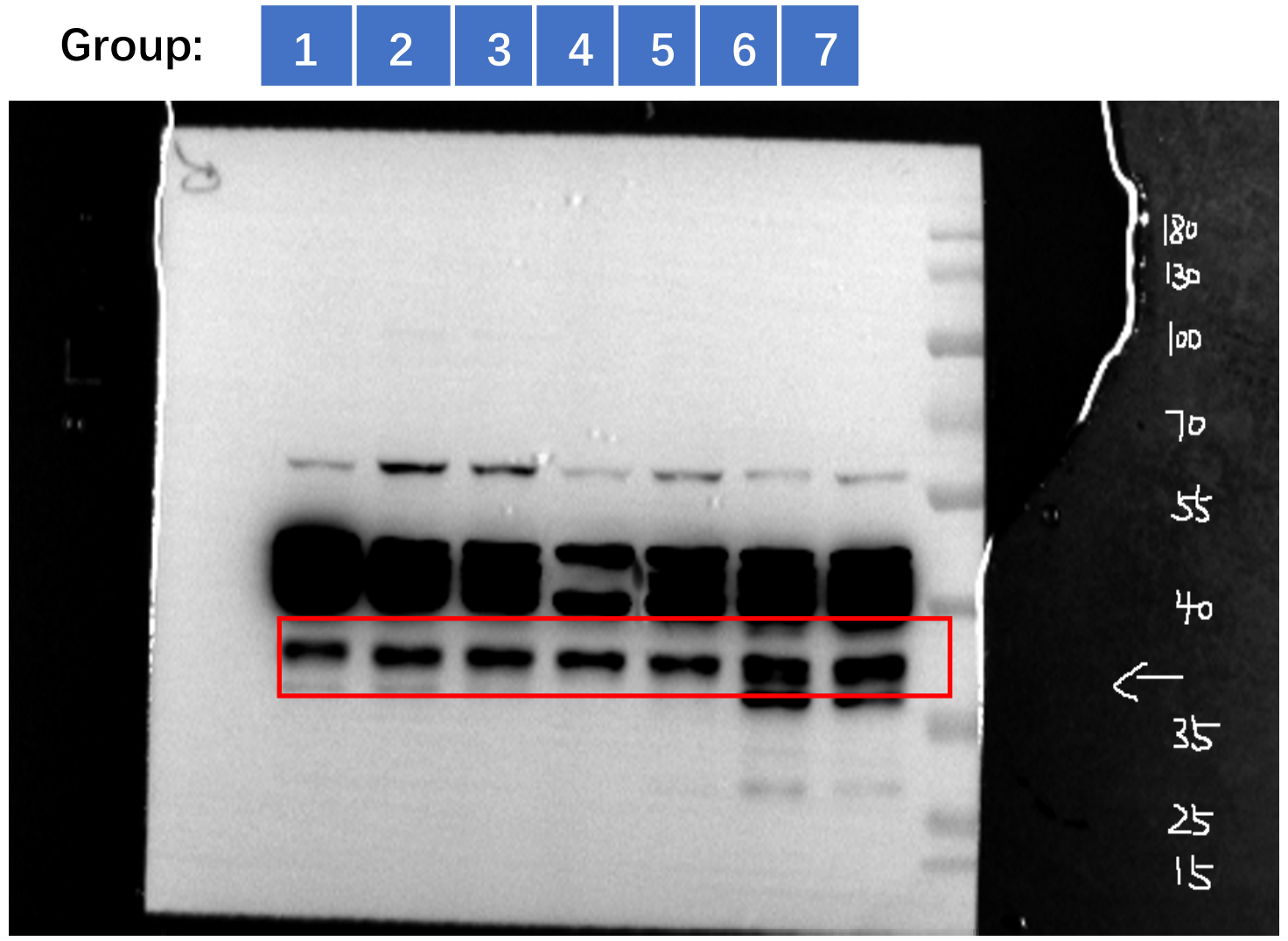

IκBα

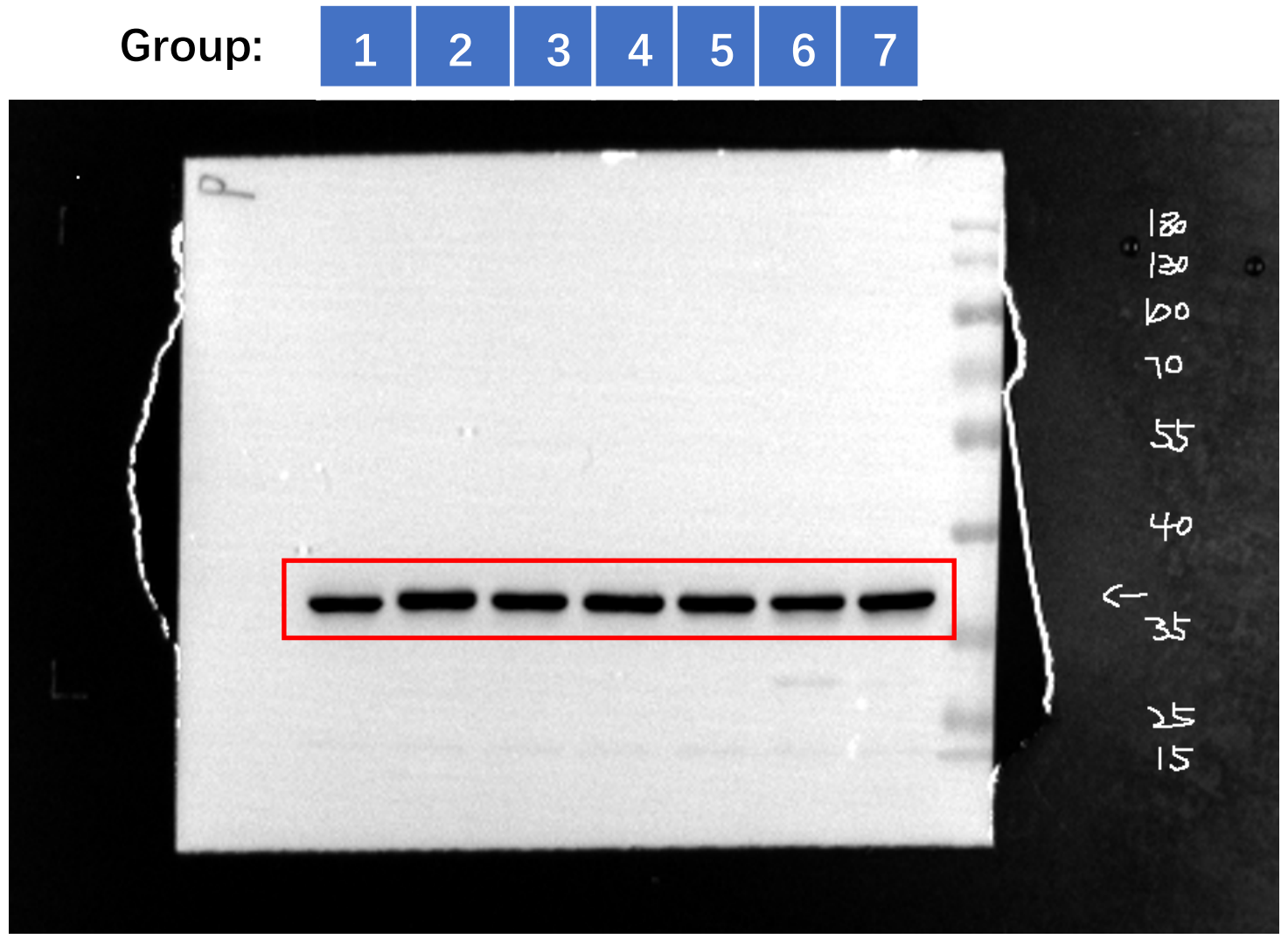

p-IκBα

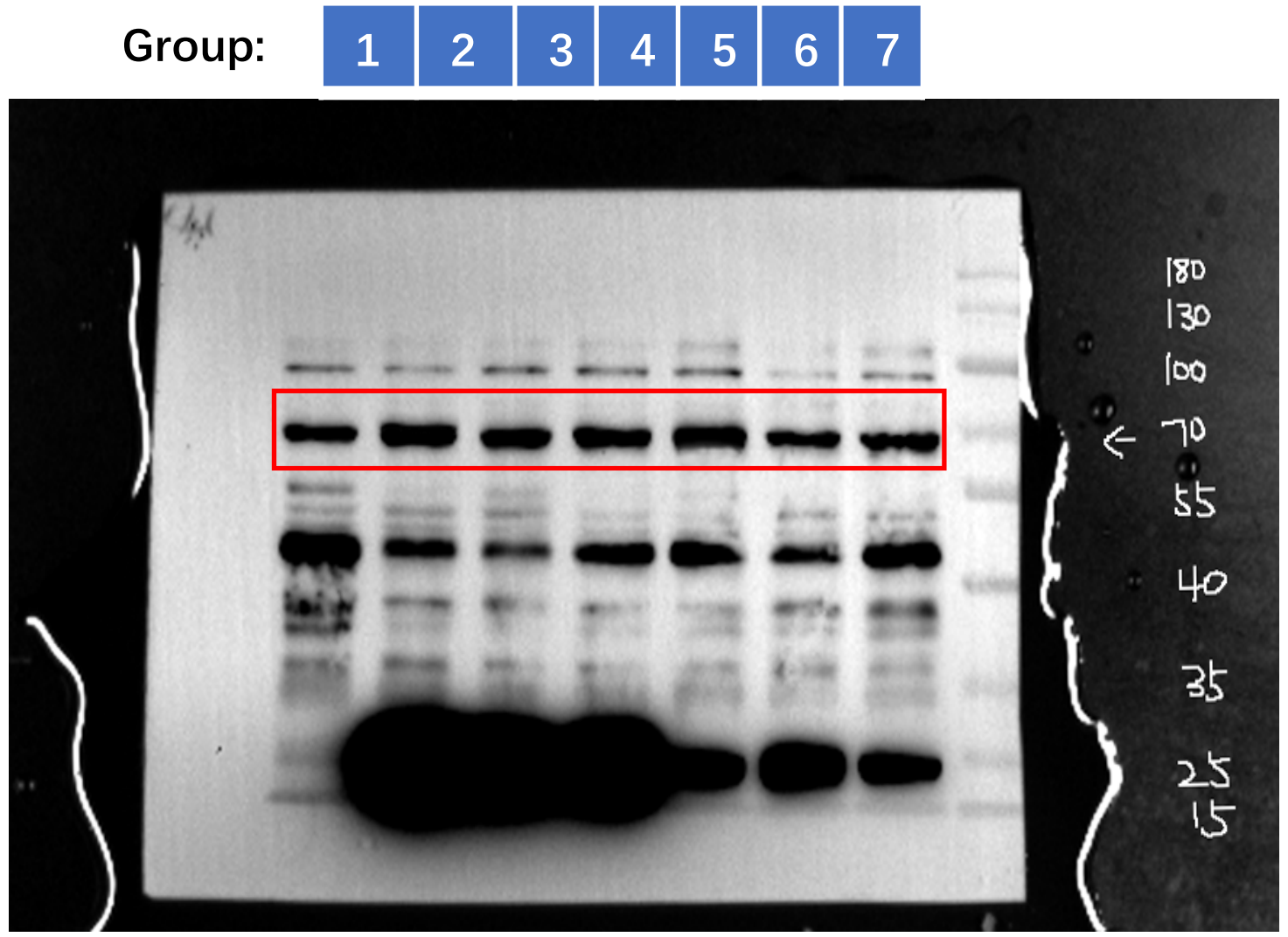

NF-κB p65

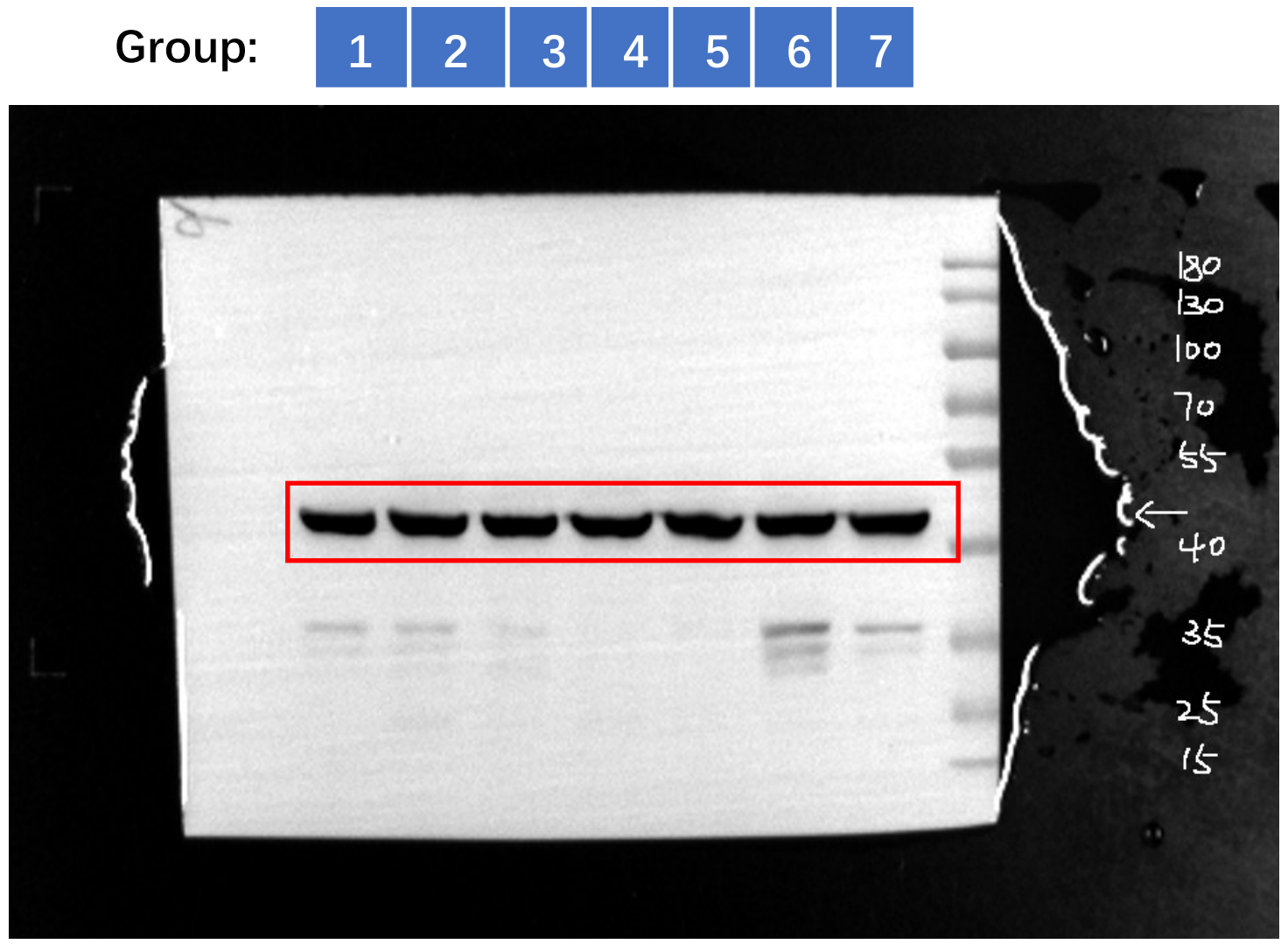

β-actin

Group:
(1) Control;
(2) OVA model;
(3) Dexamethasone (2.5 mg/kg/day);
(4) Loratadine (2 mg/kg/day);
(5) Quercetin (20mg/kg/day)
(6) Quercetin (40mg/kg/day).
(7) Quercetin (80 mg/kg/day).

Raw_WB_Images_Fig6C
