# Supplementary material for: Quercetin modulates mast cell activation and the IL-33/ST2/NF-κB axis in allergic rhinitis
Source: Sci Rep. 2026 Jun 14;16:27159. doi: 10.1038/s41598-026-57981-6 (PMC13526780; doi:10.1038/s41598-026-57981-6)
Supplement: Supplementary file 2 [file 41598_2026_57981_MOESM2_ESM.docx]

**Quercetin Modulates Mast Cell Activation and the IL-33/ST2/NF-κB Axis in Allergic Rhinitis**

Yi Xiao¹,²,³,⁴,⁵†, Meihua Xu⁴,⁵†, Li Zhong⁴,⁵, Feng Lin⁴,⁵, Minjie Yu⁴,⁵, Huiwen Luo⁴,⁵ and Chang Lin¹,²,³*

Author details

^1^Department of Otorhinolaryngology Head and Neck Surgery, The First Affiliated Hospital of Fujian Medical University, Fuzhou, China

^2^Department of Otorhinolaryngology Head and Neck Surgery, National Regional Medical Center, Binhai Campus of The First Affiliated Hospital of Fujian Medical University, Fuzhou, China

^3^Fujian Institute of Otolaryngology, The First Affiliated Hospital of Fujian Medical University, Fuzhou, China

^4^Department of Otorhinolaryngology Head and Neck Surgery, Nanping First Hospital Affiliated to Fujian Medical University, Nanping, China

^5^Nanping Institute of Otolaryngology, Nanping First Hospital Affiliated to Fujian Medical University, Nanping, China

†Yi Xiao and Meihua Xu contributed equally to this work.

*Corresponding author Chang Lin: ent_doctor@fjmu.edu.cn,[linc301@sina.com](mailto:linc301@sina.com)

**Abstract**

**Objective:** Allergic rhinitis (AR) is driven by immunoglobulin E (IgE)-mediated mast cell (MC) activation, leading to degranulation and type 2 cytokine release. Quercetin has shown anti-allergic effects, but its precise mechanism in AR remains incompletely understood.

**Methods:** Quercetin's impact on interleukin-33 (IL-33)-stimulated rat basophilic leukemia-2H3 (RBL-2H3) cells was assessed using both direct IL-33 exposure and IgE-mediated activation via anti-dinitrophenyl (DNP)-IgE sensitization and DNP- conjugated human serum albumin (HSA) challenge. Cytokine secretion, degranulation, apoptosis, and expression of IL-33 /suppression of tumorigenicity 2 (ST2)/nuclear factor kappa-light-chain-enhancer of activated B cells (NF-κB) pathway components were assessed using enzyme-linked immunosorbent assay (ELISA), Western blotting, and reverse transcription quantitative polymerase chain reaction (RT-qPCR). A mouse model of AR induced by ovalbumin (OVA) was employed to assess in vivo efficacy. Symptom scores, inflammatory cytokine levels, nasal mucosal histology, and key signaling molecule expression were analyzed to confirm quercetin's protection.

**Results:** Quercetin significantly suppressed IL-33-induced cytokine production, degranulation, and apoptosis in vitro; these effects were partially reversed by ST2 overexpression. In vivo, quercetin alleviated AR symptoms, reduced serum T helper 2 (Th2) cytokines, OVA-specific IgE (OVA-sIgE), and IL-33 levels, improved histopathology, and downregulated IL-33/ST2/NF-κB pathway proteins in nasal tissues.

**Conclusion:** Our findings demonstrate quercetin's anti-allergic effects against AR, **which are likely mediated through** suppression of mast cell activation and inhibition of the IL-33/ST2/NF-κB signaling cascade.

**Keywords:** Quercetin; Degranulation; IL-33/ST2 pathway; RBL-2H3 cells; Allergic rhinitis

1. **Introduction**

Allergic rhinitis (AR) represents a widespread chronic inflammatory condition affecting the nasal mucosa, with an estimated prevalence of 10–20% worldwide, creating substantial burdens for healthcare systems and socioeconomic frameworks. The clinical presentation of AR encompasses manifestations including nasal blockage, sneezing episodes, rhinorrhea, and nasal pruritus, significantly compromising patients' quality of life^1–3^. Contemporary therapeutic approaches encompass allergen elimination, antihistamine medications, corticosteroid treatments, and allergen-specific immunotherapy ^2,4,5^. However, these interventions frequently exhibit restricted effectiveness, are associated with adverse reactions, or fail to achieve sustained disease management. For example, extended intranasal corticosteroid administration may result in mucosal dehydration, epistaxis, and occasionally, hypothalamic-pituitary-adrenal axis suppression. Antihistamines can induce sedative effects, vertigo, and anticholinergic responses, especially among elderly populations ^6,7^. Moreover, although allergen-specific immunotherapy shows promise, it requires a lengthy treatment course and carries the risk of systemic allergic reactions in certain patients ^5^. These constraints highlight the critical requirement for enhanced and safer therapeutic options.

The pathophysiological mechanisms underlying AR are fundamentally orchestrated by immunoglobulin E (IgE)-mediated hypersensitivity reactions to environmental allergens, encompassing complex interactions between diverse immune cell populations and inflammatory mediators. Mast cells (MCs) function as principal effector cells within this pathological cascade. Their activation and degranulation constitute essential elements in AR development^8,9^. RBL-2H3 cells, representing a well-characterized in vitro mast cell degranulation model, are extensively employed for investigating cellular and molecular mechanisms governing allergic responses^10,11^.

Recent research has increasingly focused on the communication pathway linking epithelial cells and MCs, specifically emphasizing the function of interleukin-33 (IL-33) secreted by compromised epithelial cells. IL-33 is predominantly synthesized by epithelial and endothelial cells following environmental stress exposure^12^. It binds to its receptor ST2 (suppression of tumorigenicity 2), triggering signaling cascades that promote Th2 cell differentiation and release of pro-allergic cytokines, thereby amplifying type 2 immune responses^13–15^. Genomic investigations have also suggested associations between key genes in the IL-33/ST2 pathway correlate with elevated susceptibility to allergic disorders, emphasizing its fundamental role in disease development and progression^16^.

In recent years, naturally derived compounds with anti-inflammatory and immunomodulatory properties have garnered significant research interest ^17^. Quercetin, a plant-derived flavonoid compound abundant in fruits and vegetables, has undergone extensive investigation for its multifaceted biological activities, including potent antioxidant effects, significant anti-allergic actions, and demonstrated anti-inflammatory properties. Clinical and experimental investigations have established that quercetin can ameliorate allergic manifestations, including sneezing and nasal pruritus, in patients with seasonal AR^18,19^. In murine AR models induced by OVA or toluene diisocyanate (TDI), quercetin has demonstrated efficacy in relieving nasal symptoms, decreasing serum histamine concentrations, and regulating T helper 1 (Th1) / T helper 2 (Th2) and regulatory T cells (Treg) / T helper 17 (Th17) immune equilibrium^20^. Despite increasing evidence supporting quercetin's anti-allergic characteristics, its exact mechanisms in AR management remain incompletely elucidated. Although quercetin is recognized for modulating various inflammatory pathways, its impact on the IL-33/ST2 signaling cascade—fundamental to AR pathogenesis—has not received specific investigation. This constitutes a significant knowledge deficiency. Elucidating whether quercetin can modulate this pathway would not only advance comprehension of its mechanism of action but also identify potential therapeutic targets. Considering the limitations of existing AR treatments, exploring quercetin's influence on the IL-33/ST2 cascade may provide innovative perspectives for developing safer, more efficacious anti-allergic therapies and supporting precision medicine advancement in allergic disease management.

This study investigated quercetin's therapeutic mechanisms in allergic rhinitis, focusing on the IL-33/ST2/NF-κB pathway. Using RBL-2H3 cells and an OVA-induced AR mouse model, we assessed its effects on mast cell degranulation, inflammatory mediator secretion, and apoptosis in vitro. Furthermore, symptom scores, inflammatory cytokine levels, nasal tissue histology, and key signaling molecule expression were analyzed to confirm quercetin's protective role in vivo. Our discoveries may provide novel understanding of the molecular mechanisms underlying quercetin's anti-allergic properties and validate its potential therapeutic development for AR treatment.

2. **MATERIALS AND METHODS**

**2.1 Reagents**

Quercetin (purity ≥ 98%, Cat# HY-18085) and loratadine (Cat# HY-17043) were purchased from MedChemExpress (NJ, USA). Ovalbumin (OVA, purity ≥ 98%, Cat# A5503), aluminum hydroxide (Cat# 239186), anti-DNP IgE antibody (Cat# D8406), and DNP-HSA(Cat# A6661) were obtained from Sigma-Aldrich (St. Louis, MO, USA). Recombinant rat IL-33 (Cat# P6317) was supplied by Beyotime Biotechnology (Shanghai, China). Dexamethasone sodium phosphate (National Drug Approval No. H42020019) was obtained from Tianyao Pharmaceutical Co., Ltd. (Hubei, China).

ELISA kits were used as follows: Histamine (HIS, Cat# E-EL-0032) and rat monocyte chemoattractant protein-1 (MCP-1, Cat# E-EL-R0633) were obtained from Elabscience Biotechnology Co., Ltd. (Wuhan, China). The rat β-hexosaminidase (β-Hex, Cat# MM-20803R2) kit was purchased from MMbio Biotechnology Co., Ltd. (Jiangsu, China). Rat tumor necrosis factor-α (TNF-α, Cat# EK382) and interleukin-6 (IL-6, Cat# EK306) kits were obtained from MultiSciences Biotechnology Co., Ltd. (Hangzhou, China). Mouse interleukin-4 (IL-4, Cat# EK204HS), interleukin-5(IL-5) (Cat# EK205HS), interleukin-13(IL-13) (Cat# EK213), and IL-33 (Cat# EK233HS-96) kits were sourced from Lianke Biotechnology Co., Ltd. (Hangzhou, China). The OVA-sIgE(Cat# MU30065) kit was purchased from Bioswamp Biotechnology Co., Ltd. (Wuhan, China).

For cell-based assays, the Annexin V-FITC/PI Apoptosis Detection Kit (Cat# E-CK-A211) and Cell Counting Kit-8 (CCK-8, Cat# E-CK-A362) were obtained from Elabscience Biotechnology Co., Ltd. (Wuhan, China). Key reagents are listed in Supplementary Table S2.

**2.2 Molecular Docking Analysis**

Molecular docking between quercetin and IL-33 or ST2 components was performed using CB-Dock2 (<http://cadd.labshare.cn/cb-dock2/>). The three-dimensional(3D) structure of quercetin (Compound ID: MOL000098) in MOL2 format was retrieved from the TCMSP database ([https://tcmsp-e.com](https://tcmsp-e.com/)). The crystal structures of human IL-33(PDB ID: 2KLL, DOI: <https://doi.org/10.2210/pdb2kll/pdb>) and ST2(PDB ID: 4KC3, DOI: <https://doi.org/10.2210/pdb4kc3/pdb>) in Protein Data Bank (PDB) format were acquired from the RCSB PDB database ([https://www.rcsb.org](https://www.rcsb.org/)).

These molecular structures were processed and analyzed to predict binding affinities and interaction sites within the docking platform. Docking simulations were conducted using CB-Dock2, which integrates cavity detection and molecular docking via AutoDock Vina to identify the most probable binding sites and predict binding affinities. A blind docking approach was applied, and each target protein was independently docked with quercetin. The docking results were evaluated based on Vina scores, with more negative values indicating stronger binding affinities. The conformation exhibiting the lowest binding energy was designated as the most favorable binding pose. The 3D docking models, binding cavities, and interaction sites were visualized and examined to assess quercetin's interaction potential with IL-33 and ST2.

**2.3 Cell Culture and Treatment, and Transfection**

RBL-2H3 cells (Cat# CL-0192) along with Minimum Essential Medium (MEM) were sourced from Procell Life Science (Wuhan, China). Cells were maintained in MEM containing 15% fetal bovine serum (FBS) and 1% penicillin-streptomycin under standard culture conditions (37°C, 5% CO₂, humidified atmosphere). Quercetin stock solutions (0.1% dimethyl sulfoxide (DMSO)) were diluted to working concentrations (1, 5, 10 µM; final DMSO ≤0.1%).

**Cell Treatment:** For IgE sensitization, RBL-2H3 cells were first exposed to anti-DNP-IgE (500 ng/mL) for 24 h. After washing 2–3 times, IgE-sensitized cells were then incubated with quercetin (at concentrations of 1, 5, or 10 µM) for an additional 24 h, followed by treatment with DNP-HSA (500 ng/mL) and/or recombinant rat IL-33 (50 ng/mL) for 6 h.

**Transfection:** ST2 overexpression was performed in 6-well plates by transfecting cells with the pcDNA3.1-ST2 plasmid (encoding Rattus norvegicus interleukin-1 receptor-like 1 (IL-1RL1); YouBIO, Hunan, Cat# G142887) and A total of 4 μg of plasmid DNA was mixed with 10 μL TransIntro® EL Transfection Reagent (TransGen, Beijing, FT201) in serum-free medium to form complexes, according to the manufacturer's protocol. Transfection efficiency, assessed in parallel using a GFP-expressing plasmid, was consistently greater than 70%. Cells were harvested for subsequent analysis 24 hours post-transfection.

**2.4 Cell Viability**

For viability testing, RBL-2H3 cells (1×10⁴/well, 96-well plate) were treated with quercetin (0.1–80 µM) for 24 or 48 h. Subsequently, 10 µL of CCK-8 reagent was added to each well, and the optical density (OD) at 450 nm was measured using a microplate reader (Multiskan FC, Thermo Scientific).

**2.5 Cytokine Measurement**

RBL-2H3 cells (1×10⁴ cells/well in a 96-well plate) were pretreated with quercetin (1, 5, or 10 µM) for 24 h, followed by stimulation with IL-33 (50 ng/mL) for an additional 6 h. Supernatant cytokine levels (MCP-1, IL-13, TNF-α, IL-6) were measured by ELISA (450 nm absorbance; Multiskan FC microplate reader).

**2.6 Cell Proliferation Assays**

IgE-sensitized RBL-2H3 cells (1×10⁴ per well in a 96-well plate) were treated as described in Section 2.3. Proliferation was measured identically using the CCK-8 protocol.

**2.7 Cell Apoptosis Assay**

For apoptotic assessment, IgE-sensitized RBL-2H3 cells (5×10⁵ per well in a 6-well plate) were treated following the same exposure conditions as described in Section 2.3. Cells were then resuspended in 500 µL binding buffer with 5 µL Annexin V-FITC and 5 µL PI (20 min, RT, dark). Data were acquired via flow cytometry (NovoCyte 1030, ACEA Biosciences).

**2.8 Degranulation Assay**

IgE-sensitized RBL-2H3 cells (1×10⁴ per well in a 96-well plate) were treated as described in the cell treatment section. Histamine and β-hexosaminidase in supernatants were assayed by ELISA. The β-hexosaminidase release rate was derived as: % Release = [(OD_stimulated - OD_unstimulated) / (OD_Triton - OD_unstimulated)] × 100.

**2.9 RT-qPCR**

Total RNA extraction was performed using Trizol Plus columns (Yali Biotechnology, China). Reverse transcription (1 µg RNA) was carried out using ABScript Neo RT Master Mix with gDNA Remover (ABclonal). qPCR reactions (20 µL) were conducted on QuantStudio 3 (Applied Biosystems) and contained: 10 µL BrightCycle SYBR Green Mix (Yali), 0.4 µL each primer (10 µM), 1 µL cDNA, and sterile nuclease-free water. Cycling conditions: 37°C for 2 min Uracil-DNA Glycosylase (UDG) activation; 95°C for 3 min; 40 cycles of [95°C for 5 s → 60°C for 30 s]. mRNA levels were normalized to β-actin (2⁻ΔΔCt method). Primer sequences: *Il1rl1* (Rat): Forward: 5′-CGGAGAACGGAACCAATTACATA-3′; Reverse: 5′-GGACAGCAACGAACTGAGAG-3′. *β-actin* (Rat): Forward: 5′-TGAACCCTAAGGCCAACCGT-3′; Reverse: 5′-GTACGACCAGAGGCATACAGGGATA-3′.

**2.10 Animals**

Female BALB/c mice (6–8 weeks, 20 ± 2 g) were supplied by SPF Biotechnology (Suzhou, China). All mice were housed in SPF facilities with controlled temperature (23 ± 2°C), humidity (55 ± 5%), and 12-h light/dark cycles. They had unrestricted access to food and water. Throughout the study, all efforts were made to minimize suffering. The mice were monitored daily for signs of distress, and environmental enrichment was provided.  For endpoint procedures, mice were deeply anesthetized with an intraperitoneal injection of sodium pentobarbital (40 mg/kg), euthanized by cervical dislocation, and death was confirmed prior to tissue harvest. All animal experiments were performed in accordance with the National Institutes of Health Guide for the Care and Use of Laboratory Animals (8th edition) and approved by the Institutional Animal Care and Use Committee of Nanping First Hospital Affiliated to Fujian Medical University (Approval No. NPSY-202412-003).

**2.11 Animal Treatment Protocol**

Mice were randomized to seven groups (6 mice per group) using a computer-generated random number table to ensure unbiased allocation: (1) Control;(2) OVA model;(3) Dexamethasone (2.5 mg/kg/day);(4) Loratadine (2 mg/kg/day);(5–7) Quercetin (20/40/80 mg/kg/day).The experimental protocol was based on previously established OVA-induced allergic rhinitis mouse models^20–23^ and quercetin dosing regimens reported in the literature ^20,24^.

**Sensitization (days 0, 7, 14):** Intraperitoneal injection of OVA (50 µg) with aluminum hydroxide (1 mg) in a total volume of 200 µL.

**Treatment (days 15–21):** Daily oral administration of quercetin (vehicle: 45% PEG300, 5% Tween-80, 50% saline), dexamethasone, or loratadine at the respective dosages; the model group received vehicle only.

**OVA challenge (days 22–31):** Daily intranasal challenge with OVA (500 µg in 20 µL; 25 mg/mL).

**Symptom scoring:** Post-challenge (days 22–31), sneezing and nasal rubbing events were recorded for 10 minutes by blinded observers.

**Sample collection (day 32):** 24 hours post-final challenge, mice were anesthetized (sodium pentobarbital, 40 mg/kg i.p.). Blood was collected via the retro-orbital vein, and the mice were then euthanized by cervical dislocation. Nasal lavage fluid (NLF) and nasal mucosal tissues were harvested for analysis.

**2.12 Serum and Nasal Lavage Fluid（NLF） Preparation**

**For Serum Preparation:** Clotted blood (**room temperature（**RT）, 1 h) was centrifuged (3500 rpm, 10 min, 4°C).

For Nasal Lavage Fluid (NLF) Preparation: Mice were subjected to nasal lavage with ice-cold  phosphate-Buffered Saline（PBS） (1 mL/mouse, 4°C) post-euthanasia. The supernatant was collected after centrifugation (1200 rpm, 10 min).

Both serum and NLF supernatant were stored at −80°C for cytokine assays.

**2.13 Histopathological analysis**

Nasal tissues underwent the following steps: Fixation: 4% paraformaldehyde (PFA) (24 h); Decalcification: 0.5 M ethylenediaminetetraacetic acid (EDTA) (4 weeks, RT); Paraffin embedding & sectioning: 5 μm; Staining: hematoxylin and eosin (H&E) (histology), periodic acid-Schiff (PAS) (goblet cells; reagents from Servicebio, Wuhan).

For quantitative histopathology**:** Six randomly selected regions per sample were analyzed at ×400 magnification by a pathologist blinded to the group assignments Epithelial thickness was measured using ImageJ software. For goblet cell quantification, a defined segment of the nasal epithelium was selected; its length was measured, and goblet cells within the segment were manually counted. The density of goblet cells was expressed as cells per 1 μm.

**2.14 Western blot analysis**

Protein Extraction: Radioimmunoprecipitation assay (RIPA) lysis buffer (with phenylmethylsulfonyl fluoride (PMSF) + protease/phosphatase inhibitor cocktail (Beyotime, Shanghai, China)) on ice. Quantification: bicinchoninic acid (BCA) kit (Saiguo Biotech Co., Ltd., Guangzhou, China). Electrophoresis: 40 μg protein/lane, 12% sodium dodecyl sulfate-polyacrylamide gel electrophoresis (SDS-PAGE) Proteins were then transferred onto polyvinylidene difluoride (PVDF) membranes (Millipore, IPVH00010, Billerica, MA, USA).Membrane Processing: Membranes were blocked with 5% non-fat milk/  Tris-buffered saline with Tween 20 (TBST) (2 h, RT) and subsequently incubated with the following primary antibodies (4°C overnight):ST2 (1:1000, Proteintech, 11920-1-AP, Wuhan, China), Myeloid differentiation primary response 88（MyD88） (1:1000, ZenBio, #340629), nuclear factor of kappa light polypeptide gene enhancer in B-cells inhibitor, alpha (IκBα) (1:1000, ZenBio, #380682), phosphorylated IκBα (p-IκBα) (Ser32/36, 1:500, ZenBio, #340776), nuclear factor kappa-light-chain-enhancer of activated B cells p65 (NF-κB p65) (1:500, ZenBio, #R25150),β-actin (1:3000, ZenBio, #200068).After washing, membranes were incubated with horseradish peroxidase (HRP)-conjugated anti-rabbit/mouse secondary antibodies (1:4000, ZenBio; 2 h, RT). Protein bands were visualized using enhanced chemiluminescence (enhanced chemiluminescence (ECL), ZenBio) and visualized on a ChemiScope Touch Imaging System (Clinx, Shanghai, China). Densitometric analysis was performed with ImageJ software. After background subtraction, target protein levels were normalized to β-actin, whose stable expression across groups was confirmed (Supplementary Fig. S2). Data are expressed as fold change relative to the control.

3. **Statistical analysis**

Data are shown as mean ± standard deviation (SD). Statistical testing was performed using GraphPad Prism 9.0 (GraphPad Software Inc., La Jolla, CA, USA). After assessing normality (Shapiro–Wilk test) and homogeneity of variances (Brown–Forsythe test), the appropriate test was selected: ordinary one‑way ANOVA, repeated‑measures ANOVA, or Kruskal–Wallis test. Following a significant ANOVA result, multiple comparisons were corrected using Dunnett’s test (vs. a single control), Tukey’s test (all pairwise comparisons), or FDR control via the two‑stage step‑up method of Benjamini, Krieger and Yekutieli, as applicable. Effect sizes are reported as mean differences with 95% confidence intervals where appropriate. A p‑value or FDR q‑value < 0.05 was considered statistically significant. Detailed experimental designs and statistical parameters for each figure are provided in Supplementary Table S1...

4. **Results**

**4.1 Molecular Docking Analysis**

Molecular docking with CB-Dock2 assessed the potential binding interactions between

Fig 1 **Quercetin exerts anti-inflammatory effects on IL-33-Induced RBL-2H3 cells.**

(a)Chemical structure of Quercetin.(b)Molecular docking models and predicted binding affinities (Vina scores) of Quercetin with IL-33 and ST2.(c)Cell viability assessed by CCK-8 assay after treatment with Quercetin (0.1-80 µM) for 24 or 48 hours.(d-e)Cytokine levels (IL-6, IL-13, TNF-α, MCP-1) in cell supernatant measured by ELISA. ST2-OE: ST2-overexpressing cells. Data are presented as mean ± SD (n = 3). Statistical comparisons were performed against the Control group (*), the IL-33-treated group (#), and the Vector-NC group (^). Significance levels: *, #, or ^ p < 0.05; **, ##, or ^^ p < 0.01; ***, ###, or ^^^ p < 0.001.

key components of the IL-33/ST2 signaling pathway and quercetin (Fig 1a). Quercetin was docked separately against human IL-33 (PDB ID: 2KLL) and ST2 (PDB ID: 4KC3). The results demonstrated potential binding interactions with both targets. The predicted binding affinity scores (kcal/mol) were -49.4 for IL-33 and -9.2 for ST2 (Fig 1b). Among the complexes tested, the ST2-quercetin and IL-33-quercetin complexes exhibited lower (more negative) binding energies, which might imply a tendency for stronger interactions with these targets.

**4.2 Quercetin Inhibits IL-33-Induced Cytokine Secretion**

To establish the cytotoxic threshold of quercetin, RBL-2H3 cells were treated with a range of quercetin concentrations from 0.1 to 80 μM. Cell viability assays indicated significant cytotoxicity at concentrations ≥20 μM (Fig 1c). Therefore, concentrations of 1, 5, and 10 μM were selected for the following experiments.

Upon IL-33 stimulation, RBL-2H3 cells showed elevated secretion of IL-6, IL-13, TNF-α, and MCP-1. Quercetin treatment dose-dependently suppressed the secretion of these pro-inflammatory cytokines (Fig 1d). Furthermore, under IL‑33 stimulation with 5 µM quercetin, ST2‑overexpressing cells exhibited a partial rescue of cytokine production (IL‑6, TNF‑α, MCP‑1) compared to vector‑NC cells (Fig 1e) These results suggest that ST2 plays an important role in IL‑33‑driven inflammation, and that quercetin’s inhibitory effect is at least partially mediated through this receptor.

**4.3 Quercetin Inhibits Degranulation and Apoptosis**

To induce mast cell degranulation, RBL-2H3 cells underwent IgE-mediated activation through anti-DNP-IgE sensitization followed by DNP-HSA challenge, either alone or in combination with IL-33 supplementation. Viability assessment revealed that cellular integrity was preserved under both IgE-mediated cross-linking and IL-33 stimulation conditions, as demonstrated in (Fig 2a). However, degranulation markers, including histamine and β-hexosaminidase, were significantly increased following stimulation and were markedly suppressed

Fig 2 **Quercetin attenuates IL-33-induced degranulation and apoptosis in activated RBL-2H3 cells via the IL-33/ST2/NF-κB signaling pathway.**

(a) Cell viability was measured by CCK-8 assay. (b) Histamine concentration and β-hexosaminidase release rate by ELISA. (c-d) Apoptosis by flow cytometry. The gating strategy is detailed in Supplementary Figure S1. (e-f) Protein levels of ST2, MyD88, p-IκBα/IκBα, NF-κB p65 by western blotting (β-actin as control). IgE group: Sensitized with anti-DNP-IgE and challenged with DNP-HSA. Data are presented as mean ± SD (n = 3). Lanes were cropped from different gels (each antibody was detected on a separate gel) and grouped for comparison; black lines indicate boundaries between gels. All samples were derived from the same experiment, and gels were processed in parallel. Full-length, uncropped blots with visible membrane edges are presented in Supplementary Figure 1. Statistical comparisons were performed against the Control group (*), the IgE group (#), IgE+IL-33-treated group (^). Significance levels: *, #, or ^ p < 0.05; **, ##, or ^^ p < 0.01; ***, ###, or ^^^ p < 0.001.

by quercetin (Fig 2b). During the simulated degranulation process, flow cytometric analysis revealed that co-treatment with DNP-IgE and DNP-HSA significantly enhanced apoptosis in RBL-2H3 cells, irrespective of IL-33 stimulation. This response was markedly attenuated upon quercetin treatment (Fig 2c-d).

**4.4 Quercetin Attenuates Mast Cell Degranulation via IL-33/ST2/NF-κB Suppression**

Western blot analysis showed that during the degranulation process in RBL-2H3 cells, the expression of ST2, MyD88, and NF-κB (p65) was upregulated, and the p-IκBα/IκBα ratio was increased. IL-33 stimulation exacerbated this process, suggesting the activation of the IL-33/ST2/NF-κB signaling pathway (Fig 2e-f). Quercetin treatment effectively down-regulated these key signaling molecules, suggesting suppression of pathway activation.

To further confirm that the anti-inflammatory effects of quercetin are mediated via this pathway, RBL-2H3 cells were transfected with a pcDNA-ST2 plasmid to induce ST2 overexpression. The successful establishment of overexpression was verified using RT-qPCR and Western blot assays (Fig 3a-c). ST2 overexpression slightly reduced cell viability (Fig 3d) and enhanced mast cell degranulation and apoptosis, even in the presence of quercetin (Fig 3e-f). Consistently, Western blot analysis demonstrated that ST2 overexpression significantly upregulated the expression of ST2, MyD88, and NF-κB (p65), while increasing the p-IκBα/IκBα ratio. Although quercetin still downregulated these proteins, its inhibitory effects were markedly attenuated (Fig 3h), indicating that ST2 is an important mediator of quercetin’s anti‑inflammatory action..

Fig 3 **Quercetin reversed the enhanced degranulation and apoptosis induced by ST2 overexpression in** RBL-2H3 **cells.**

(a-b) ST2 protein levels by western blotting. (c) ST2 mRNA levels by qRT-PCR. (d) Cell viability by CCK-8 assay. (e) histamine concentration and β-Hexosaminidase release rate by ELISA. (f-g) Apoptosis by flow cytometry. (h) Protein levels of ST2, MyD88, IκBα, p-IκBα, NF-κB (p65) by western blotting (β-actin as control). Full-length, uncropped blots with visible membrane edges are presented in Supplementary Figure 1. Data are presented as mean ± SD (n = 3). Statistical significance: *p < 0.05, **p < 0.01, ***p < 0.001. among the compared groups.

**4.5 Quercetin Attenuates OVA-Induced Allergic Rhinitis and Th2 Inflammation**

To test quercetin’s in vivo effects, an ovalbumin (OVA)-induced allergic rhinitis (AR) mouse model was established (Fig 4a). Behavioral observations demonstrated that AR mice displayed a significantly higher frequency of sneezing and nasal rubbing compared to control mice (Fig 4b-c). Treatment with quercetin (20/40/80 mg/kg) significantly reduced both nasal symptoms.

To evaluate systemic and local type 2 immune responses, serum levels of IL-4, IL-5, IL-13 (known as Th2 signature cytokines), IL-33, and OVA-specific IgE were quantified. Quercetin significantly decreased these cytokines and OVA-sIgE versus the AR group (Fig 4d-g). Quercetin also markedly decreased IL-33 levels in both serum and nasal lavage fluid (NLF) (Fig 4h-i), indicating its potential to suppress upstream allergic signaling.

Fig 4 **Quercetin Attenuates Allergic Symptoms and Th2-Mediated Inflammation in OVA-Induced AR Mice.**

(a) Experimental timeline. (b, c) Sneezing and nasal rubbing counts (10 min, days 22–32). (d–g) Serum IL-4, IL-5, IL-13, OVA-sIgE levels. (h–i) IL-33 levels in serum and NLF. Data are presented as mean ± SD (n = 6). Statistical comparisons were performed against the Control group (*) and the Model group (#). Significance levels: * or # p < 0.05, ** or ## p < 0.01, *** or ### p < 0.001.

**4.6 Quercetin Reverses OVA-Induced Histopathological Alterations in Nasal Mucosa**

To further assess the protective effects of quercetin on nasal tissue architecture, H&E and PAS staining were used for histological analysis. In AR mice, nasal mucosal tissues exhibited typical pathological features, including mucosal thickening, submucosal inflammatory cell infiltration, goblet cell hyperplasia, and enlarged glandular areas (Fig 5). These changes were substantially ameliorated following treatment with quercetin, especially at the 40 mg/kg dose. In contrast, loratadine treatment exerted only a marginal effect on mucosal remodeling (Fig 6a). PAS staining further confirmed that quercetin significantly reduced the density of goblet cells in the nasal epithelium, indicating its efficacy in suppressing mucus hypersecretion (Fig 6b).

Fig 5 **Quercetin restores histopathological changes in the nasal mucosa of OVA-induced AR mice.**

H&E and PAS staining of nasal mucosa from mice in each group (40× and 400× magnification, n = 3/group). Indicators: Red arrows = goblet cells; yellow arrows = epithelial thickness measurement; green arrows = inflammatory cell infiltration.)

**4.7 Quercetin Suppresses IL-33/ST2/NF-κB Pathway in Nasal Tissue**

Fig 6 **Quercetin alleviates IL33/ST2/NF-κB pathway in OVA-induced AR mice.**

**(a)**  Mucosal epithelial thickness. **(b)** Goblet cell density. **(c)** Representative western blot images of proteins in the IL-33/ST2/NF-κB pathway. Full-length, uncropped blots with visible membrane edges are presented in Supplementary Figure 1. **(d-f)** Quantitative analysis of protein levels of ST2, p-IκBα/IκBα, and NF-κB (p65), normalized to β-actin.
Data are presented as mean ± SD (n = 6 for a, b; n = 3 for d-f). Statistical comparisons were performed against the Control group (*) and the Model group (#). Significance levels: * or # p < 0.05, ** or ## p < 0.01, *** or ### p < 0.001.

To investigate quercetin's anti-inflammatory mechanism, we analyzed IL-33/ST2/NF-κB pathway components in nasal tissue.

Western blotting revealed that OVA-induced allergic rhinitis (AR) significantly increased nasal mucosal levels of ST2, phospho-IκBa/IκBa ratio, and NF-κB p65. However, upon treatment with quercetin, we observed a marked dose-dependent downregulation of these signaling molecules (Fig. 6C-D). Dexamethasone and loratadine comparably inhibited pathway activation, suggesting its relevance to AR pathogenesis.

5. **Discussion**

The present study provides evidence that quercetin exerts potent anti-inflammatory and anti-allergic effects in allergic rhinitis (AR), both in vitro and in vivo. Our findings not only validate its therapeutic efficacy but also suggest a potential mechanism of action, particularly pointing to the involvement of the IL-33/ST2 signaling via the NF-κB pathway.

This mechanistic insight carries significant translational weight. The IL-33/ST2 pathway is a clinically relevant driver of human AR, and our data implicate it as a primary target for quercetin. This suggests that quercetin may act, at least in part, as a modulator of this disease-critical pathway.

**5.1 Molecular docking suggests quercetin may interact with the IL-33/ST2 pathway**

Quercetin is a polyphenolic flavonoid with a planar structure that facilitates hydrogen bonding and π-π stacking interactions with multiple protein targets. Its ability to bind both IL-33 and ST2 is consistent with its multi-targeting nature. Molecular docking analysis indicated that quercetin has the potential to bind directly to IL-33 or its receptor ST2, which could interfere with complex formation or subsequent signaling events. These computational results offer a hypothetical mechanism for its immunomodulatory activity, though experimental validation is required.

**5.2 Quercetin Regulates MCs Activation**

Mast cells (MCs) serve as central mediators of allergic inflammation, initiating immune responses upon activation via Fc epsilon RI (FcεRI) -bound IgE crosslinking. MC activation—characterized by inflammatory cytokine release and degranulation—represents a critical process in allergic rhinitis (AR) pathogenesis. This cascade triggers the secretion of multiple inflammatory mediators, including histamine and type 2 cytokines, which collectively contribute to hallmark symptoms of allergic rhinitis.^25–27^. Among mast cell surface receptors, ST2 (the IL-33 receptor) holds a crucial position. ST2 can sense IL-33 released from epithelial cells. Once IL-33 binds to ST2, it triggers a series of intracellular signaling events via NF-κB and mitogen-activated protein kinase (MAPK) pathways. As a result, allergic inflammation is amplified^26,28,29^.

BL-2H3 cells—a widely accepted in vitro model—were used to simulate mast cell activation, particularly degranulation, as observed in allergic reactions. These cells closely resemble mucosal-type mast cells in terms of morphology, surface receptor expression (e.g., FcεRI and ST2), and mediator release ^10,30^. Moreover, they exhibit strong responsiveness to both IL-33 and IgE-mediated stimulation, making them a reliable model for investigating the mechanisms underlying allergic responses.

In this study, quercetin significantly suppressed IL-33-induced production of key pro-inflammatory cytokines, as shown in Figure 1. In addition, quercetin effectively inhibited degranulation in RBL-2H3 cells following IgE sensitization and FcεRI crosslinking. Notably, co-stimulation with IL-33 further enhanced degranulation, which was also markedly attenuated by quercetin, indicating its ability to modulate mast cell responses in both innate and adaptive immune settings. Interestingly, quercetin also reduced IL-33-induced mast cell apoptosis, suggesting a potential cytoprotective effect that merits further mechanistic investigation. Collectively, our findings underscore the broad immunomodulatory properties of quercetin on mast cell function, highlighting its potential in mitigating allergic inflammation at multiple levels. Existing evidence establishes quercetin's capacity to suppress cytokine secretion and inhibit NF-κB activation across human and murine mast cell models ^31–33^.

**5.3 Quercetin Suppresses the IL-33/ST2/NF-κB Signaling Pathway**

Th2-dominant inflammation in allergic conditions is strongly driven by the well-established IL-33/ST2 signaling pathway. Upon IL-33 binding, ST2 activates downstream signaling cascades—particularly NF-κB—via phosphorylation of IκBα, promoting the transcription of cytokines and chemokines associated with Th2 polarization and eosinophilic recruitment^14,28,34,35^.

Our findings demonstrate that quercetin suppresses ST2 expression in RBL-2H3 cells, evident at the translational (protein) levels (Fig 2e). Additionally, quercetin administration led to a marked reduction in IκBα phosphorylation and NF-κB p65 expression, thereby demonstrating the inhibition of downstream inflammatory signaling pathways. Further supporting this mechanism, ST2 overexpression in RBL-2H3 cells significantly enhanced the release of inflammatory cytokines upon IL-33 stimulation, as well as degranulation resulting from co-stimulation with IL-33 and IgE. Notably, ST2 overexpression partially reversed quercetin-mediated suppression of NF-κB activation (Fig. 3h). These findings suggest that quercetin’s inhibitory effect on NF-κB is at least in part dependent on ST2. Given the substantial body of clinical and animal evidence demonstrating upregulation of the IL-33/ST2 axis in several common type 2 inflammatory diseases—such as asthma, AR, and atopic dermatitis ^21,36,37^ —our findings further reinforce the potential of this pathway as a viable therapeutic target in allergic conditions. Interestingly, a non-linear dose–response relationship was observed (Fig 2e-f), wherein lower concentrations of quercetin (1 μM) resulted in greater suppression of ST2 expression than higher doses. This suggests a possible competitive or allosteric inhibitory mechanism, which warrants further investigation through binding assays or structural modeling approaches.

**5.4 Quercetin Attenuates Nasal Inflammation and Histopathological Changes in an AR Mouse Model**

To validate these in vitro findings, we employed a well-characterized OVA-induced AR mouse model, which closely mimics human AR pathology. Repeated intranasal challenges induced classic AR symptoms, including sneezing and nasal rubbing, which were significantly attenuated by oral quercetin administration. Histopathological analysis demonstrated that quercetin attenuated nasal mucosal thickening, goblet cell hyperplasia, and submucosal inflammatory cell infiltration. ELISA assays further confirmed reduced concentrations of IL-33, OVA-specific IgE, and type 2 cytokines in both serum and nasal lavage fluid, indicating suppression of systemic and local Th2-mediated immune responses.

**5.5 Quercetin Attenuates the IL-33/ST2-NF-κB Cascade in Nasal Mucosal Tissue**

Western blot analysis of nasal tissues provided supporting in vivo evidence of quercetin's mechanism of action, showing downregulation of ST2 and NF-κB p65, as well as a decreased phospho-IκBα/IκBα ratio in the downstream signaling pathway. The significant correlation between these molecular changes and improved clinicopathological manifestations underscores the therapeutic value of inhibiting the IL-33/ST2-NF-κB cascade. Moreover, quercetin's effects were comparable to those of loratadine and dexamethasone, standard anti-allergic therapies, yet without observed toxicity or mortality. This reinforces its potential as a safe, multi-targeted agent for AR treatment. In addition to modulating the IL-33/NF-κB axis, previous studies have shown that quercetin downregulates neuropeptides such as substance P,  calcitonin gene-related peptide (CGRP), and nerve growth factor (NGF) in allergic rhinitis (AR) models, restores Th1/Th2 balance, and inhibits epithelial damage and mucosal remodeling ^20,38^. similarly, other flavonoids—such as apigenin and resveratrol—have been reported to exert anti-allergic effects through the Toll-like receptor 4 (TLR4)/myeloid differentiation primary response 88 (MyD88) axis and IL-33-related signaling pathways, respectively^29,39^, suggesting a potential class effect among flavonoids. Collectively, these findings further support the therapeutic potential of quercetin in the management of allergic rhinitis.

**5.6 Limitations and Future Perspectives**

The selection of in vivo doses (20, 40, and 80 mg/kg/day) in our study warrants specific discussion. While these exceed typical human dietary intake (7-15 mg/kg/day), they are consistent with established preclinical dosing regimens necessary to achieve therapeutic plasma and tissue levels in murine models, a common practice justified by the well-documented low oral bioavailability and rapid metabolism of quercetin in rodents ^40^.Despite its well-documented anti-allergic properties, quercetin's translation into clinical practice remains constrained by several key challenges—most notably, its low oral bioavailability, which results in only a small fraction of the ingested dose reaching systemic circulation. Innovative delivery systems, such as liposomal encapsulation, polymeric nanoparticles, or prodrug strategies, should be explored to enhance its pharmacokinetic properties ^41,42^.

It is important to acknowledge the limitations of the cellular model employed in this study. Although the RBL-2H3 cell line is well-characterized and widely used as a reproducible model for investigating IgE- and IL-33–mediated mast cell activation^28,43^, it is of rodent origin. As such, its capacity to fully recapitulate the pathophysiological context of human allergic rhinitis remains limited. While this model was appropriate for the initial dissection of relevant signaling pathways, caution should be exercised when extrapolating these findings to human systems. Therefore, a critical future direction will be to validate the inhibitory effects of quercetin in primary human mast cells—such as those derived from peripheral blood—or in human mast cell lines, to better ascertain the translational relevance of our results.

Another limitation lies in the scope of degranulation markers assessed. The present study focused on histamine and β-hexosaminidase as classical indicators of mast cell degranulation. However, mast cells release a broad spectrum of bioactive mediators—including tryptase, prostaglandins, and leukotrienes—that contribute distinctly to allergic inflammation. Examining whether quercetin modulates the release or synthesis of these additional mediators represents an important avenue for future investigation. Furthermore, one limitation of our in vitro degranulation experiments is the use of the anti-DNP IgE antibody (clone SPE-7), which has been reported to contain contaminating IgG that can bind to Fcγ receptors and influence mast cell activation^44^. Moreover, the hapten carrier system (DNP-HSA) is an artificial model that may not fully reflect natural allergen-induced mast cell activation. Therefore, our quantitative degranulation results should be interpreted with caution. Future studies should employ purified IgE antibodies (e.g., IgG‑depleted SPE‑7 or alternative IgE clones) and more physiological allergens to validate these findings.

In this study, our mechanistic investigation mainly focused on the IL-33/ST2/NF-κB signaling. However, it is important to note that IL-33/ST2 signaling also involves other critical pathways, such as the phosphoinositide 3-kinase (PI3K)/AKT strain transforming (Akt)/mechanistic target of rapamycin (mTOR) and MAPK pathways^14,28,43^, which are known to contribute to allergic inflammation. Quercetin has been reported to inhibit NF‑κB activation induced by multiple stimuli, including LPS and ischemia/reperfusion injury ^24,45^. Therefore, its effect on the NF‑κB pathway is not entirely specific to IL‑33/ST2. Nonetheless, our ST2 overexpression, direct IL-33 stimulation, and in vivo ST2 downregulation data collectively indicate that the IL-33/ST2 axis is an important mediator of quercetin’s anti-allergic action. A more comprehensive analysis incorporating these signaling cascades—potentially through transcriptomic or multi-omics approaches—may further elucidate quercetin's regulatory network in AR.

6. **Conclusion**

In conclusion, this study demonstrates that quercetin exerts anti-allergic and anti-inflammatory effects by suppressing mast cell activation—evidenced by decreased cytokine secretion and degranulation—reducing apoptosis in vitro, and by alleviating nasal symptoms and histopathological changes associated with allergic rhinitis in vivo. Mechanistically, our data suggest that the therapeutic effect of quercetin appears to involve the inhibition of the IL-33/ST2 axis and its downstream NF-κB activation, thereby pointing to a disruption of key pathways that drive allergic inflammation (Fig 7). These findings highlight quercetin's therapeutic potential.

Fig 7 **Mechanism of Quercetin in Modulating Allergic Inflammation in OVA-Induced Allergic Rhinitis**

In the OVA-induced allergic rhinitis, type II inflammation is upregulated. Activated MCs release inflammatory cytokines and undergo degranulation, releasing histamine and triggering allergic inflammatory symptoms. Damaged epithelial cells release IL-33, which binds to the ST2 receptor on MCs. This interaction activates the NF-κB pathway, thereby amplifying the inflammatory response. Quercetin may exert anti-allergic inflammatory effects by modulating the IL-33/ST2/NF-κB signaling pathway.

AUTHOR CONTRIBUTIONS

**Yi Xiao**: conceptualization, data curation, formal analysis, investigation, methodology, validation, visualization, writing-original draft, writing – review and editing. **Meihua Xu**: conceptualization, data curation, and writing – original draft, writing – review and editing. **Li Zhong**: data curation, validation, writing – review and editing. **Feng Lin:** data curation, validation, writing – review and editing. **Minjie Yu:** data curation, writing – review and editing. **Huiwen Luo:** data curation, writing – review and editing. **Chang Lin:** conceptualization, project administration, supervision, writing – review and editing.

CONFLICT OF INTEREST

The authors declare that there are no conflicts of interest.

AVAILABILITY OF DATA AND MATERIALS

The datasets generated and analysed during the current study are available in the Figshare repository: <https://doi.org/10.6084/m9.figshare.30509624>.

FUNDING/ ACKNOWLEDGMENTS

This research was financially supported by the Natural Science Foundation of Fujian Province (Grant No. 2024J011605) and the Joint Fund for Science and Technology Innovation of Nanping City (Grant No. N2024LH019).

SUPPLEMENTARY MATERIAL

There is no supplementary material available for this article.

ABBREVIATIONS

- AR: Allergic rhinitis
- OVA: Ovalbumin
- IgE: Immunoglobulin E
- IL: Interleukin
- TNF-α: Tumor necrosis factor-alpha
- MCP-1: Monocyte chemoattractant protein-1
- ST2: Suppression of tumorigenicity 2
- NF-κB: Nuclear factor kappa-light-chain-enhancer of activated B cells
- IκBα: Inhibitor of NF-κB alpha
- p-IκBα:phosphorylated IκBα
- RBL-2H3: Rat basophilic leukemia-2H3
- Th2: T helper type 2
- p-IκBα: Phosphorylated IκBα
- p-p65: Phosphorylated NF-κB(p65)
- ELISA: Enzyme-linked immunosorbent assay
- WB: Western blo**t**

**Reference**

1.	Bernstein, J. A., Bernstein, J. S., Makol, R. & Ward, S. Allergic Rhinitis: A Review. *JAMA* **331**, 866–877 (2024).

2.	Wise, S. K. *et al.* International consensus statement on allergy and rhinology: Allergic rhinitis - 2023. *Int Forum Allergy Rhinol* **13**, 293–859 (2023).

3.	Bousquet, J. *et al.* Allergic rhinitis. *Nat Rev Dis Primers* **6**, 95 (2020).

4.	Klimek, L. *et al.* Current Management of Allergic Rhinitis. *J Allergy Clin Immunol Pract* **12**, 1399–1412 (2024).

5.	Creticos, P. S., Gunaydin, F. E., Nolte, H., Damask, C. & Durham, S. R. Allergen Immunotherapy: The Evidence Supporting the Efficacy and Safety of Subcutaneous Immunotherapy and Sublingual Forms of Immunotherapy for Allergic Rhinitis/Conjunctivitis and Asthma. *J Allergy Clin Immunol Pract* **12**, 1415–1427 (2024).

6.	Siddiqui, Z. A., Walker, A., Pirwani, M. M., Tahiri, M. & Syed, I. Allergic rhinitis: diagnosis and management. *Br J Hosp Med (Lond)* **83**, 1–9 (2022).

7.	Wise, S. K. *et al.* International consensus statement on allergy and rhinology: Allergic rhinitis - 2023. *Int Forum Allergy Rhinol* **13**, 293–859 (2023).

8.	Zoabi, Y., Levi-Schaffer, F. & Eliashar, R. Allergic Rhinitis: Pathophysiology and Treatment Focusing on Mast Cells. *Biomedicines* **10**, 2486 (2022).

9.	Nur Husna, S. M., Tan, H.-T. T., Md Shukri, N., Mohd Ashari, N. S. & Wong, K. K. Allergic Rhinitis: A Clinical and Pathophysiological Overview. *Front Med (Lausanne)* **9**, 874114 (2022).

10.	Falcone F. H., Wan D., Barwary N. & Sagi-Eisenberg R. RBL cells as models for in vitro studies of mast cells and basophils. *Immunol Rev* **282**, 47–57 (2018).

11.	Rana, T. S., Bansode, R. R. & Williams, L. L. Anti-Allergic and Anti-Inflammatory Signaling Mechanisms of Natural Compounds/Extracts in In Vitro System of RBL-2H3 Cell: A Systematic Review. *Cells* **13**, 1389 (2024).

12.	Molofsky, A. B., Savage, A. K. & Locksley, R. M. Interleukin-33 in Tissue Homeostasis, Injury, and Inflammation. *Immunity* **42**, 1005–1019 (2015).

13.	Cayrol, C. IL-33, an Alarmin of the IL-1 Family Involved in Allergic and Non Allergic Inflammation: Focus on the Mechanisms of Regulation of Its Activity. *Cells* **11**, 107 (2021).

14.	Sheng F. *et al.* IL-33/ST2 axis in diverse diseases: regulatory mechanisms and therapeutic potential. *Frontiers in Immunology* **16**, 1533335 (2025).

15.	Schmitz, J. *et al.* IL-33, an Interleukin-1-like Cytokine that Signals via the IL-1 Receptor-Related Protein ST2 and Induces T Helper Type 2-Associated Cytokines. *Immunity* **23**, 479–490 (2005).

16.	Wjst, M. Exome variants associated with asthma and allergy. *Sci Rep* **12**, 21028 (2022).

17.	Li, B., Dong, B., Xie, L. & Li, Y. Exploring Advances in Natural Plant Molecules for Allergic Rhinitis Immunomodulation in Vivo and in Vitro. *Int J Gen Med* **18**, 529–565 (2025).

18.	Tosca, M. A. *et al.* Lertal®, a multicomponent nutraceutical, could reduce the use of antihistamines in children with allergic rhinoconjunctivitis. *Acta Biomed* **91**, 356–359 (2020).

19.	Yamada, S., Shirai, M., Inaba, Y. & Takara, T. Effects of repeated oral intake of a quercetin-containing supplement on allergic reaction: a randomized, placebo-controlled, double-blind parallel-group study. *Eur Rev Med Pharmacol Sci* **26**, 4331–4345 (2022).

20.	Ke, X., Chen, Z., Wang, X., Kang, H. & Hong, S. Quercetin improves the imbalance of Th1/Th2 cells and Treg/Th17 cells to attenuate allergic rhinitis. *Autoimmunity* **56**, 2189133 (2023).

21.	Zhang, J. J. *et al.* Xiao-qing-long-tang ameliorates OVA-induced allergic rhinitis by inhibiting ILC2s through the IL-33/ST2 and JAK/STAT pathways. *Phytomedicine : international journal of phytotherapy and phytopharmacology* **119**, 155012 (2023).

22.	Jin, J. *et al.* Fallopia japonica Root Extract Ameliorates Ovalbumin-Induced Airway Inflammation in a CARAS Mouse Model by Modulating the IL-33/TSLP/NF-κB Signaling Pathway. *International journal of molecular sciences* **24**, (2023).

23.	Cao, J. *et al.* Effect of kaempferol on IgE-mediated anaphylaxis in C57BL/6 mice and LAD2 cells. *Phytomedicine : international journal of phytotherapy and phytopharmacology* **79**, (2020).

24.	Li, L. *et al.* Quercetin improves cerebral ischemia/reperfusion injury by promoting microglia/macrophages M2 polarization via regulating PI3K/Akt/NF-κB signaling pathway. *Biomedicine & Pharmacotherapy* **168**, 115653 (2023).

25.	Steelant, B. *et al.* Histamine and T helper cytokine–driven epithelial barrier dysfunction in allergic rhinitis. *Journal of Allergy and Clinical Immunology* **141**, 951-963.e8 (2018).

26.	Costanzo G. *et al.* Mast Cells in Allergic and Non-Allergic Upper Airways Diseases: Sentinel in the Watchtower. *Int J Mol Sci* **25**, 12615 (2024).

27.	Gelardi M., Giancaspro R., Cassano M. & Ribatti D. The underestimated role of mast cells in the pathogenesis of rhinopathies. *International Archives of Allergy and Immunology* **183**, 153–159 (2022).

28.	Nian, J. B. *et al.* Epithelial cells expressed IL-33 to promote degranulation of mast cells through inhibition on ST2/PI3K/mTOR-mediated autophagy in allergic rhinitis. *Cell cycle (Georgetown, Tex.)* **19**, 1132–1142 (2020).

29.	Nakajima, S. *et al.* Resveratrol inhibits IL-33–mediated mast cell activation by targeting the MK2/3–PI3K/Akt axis. *Scientific reports* **9**, (2019).

30.	Xu, Y., Liu, Q., Guo, X., Xiang, L. & Zhao, G. Resveratrol attenuates IL-33-induced mast cell inflammation associated with inhibition of NF-kappaB activation and the P38 signaling pathway. *Molecular medicine reports* https://doi.org/10.3892/mmr.2020.10952 (2020) doi:10.3892/mmr.2020.10952.

31.	Kempuraj, D. *et al.* Inhibitory effect of quercetin on tryptase and MCP-1 chemokine release, and histidine decarboxylase mRNA transcription by human mast cell-1 cell line. *Clinical and Experimental Allergy* **36**, 789–796 (2006).

32.	Kandere‐Grzybowska, K., Kempuraj, D., Cao, J., Cetrulo, C. L. & Theoharides, T. C. Regulation of IL‐1‐induced selective IL‐6 release from human mast cells and inhibition by quercetin. *British J Pharmacology* **148**, 208–215 (2006).

33.	Alam, S. B., Wagner, A., Willows, S. & Kulka, M. Quercetin and Resveratrol Differentially Decrease Expression of the High-Affinity IgE Receptor (FcεRI) by Human and Mouse Mast Cells. *Molecules* **27**, 6704 (2022).

34.	Li, Y.-Q. *et al.* IL-33/ST2 axis promotes the inflammatory response of nasal mucosal epithelial cells through inducing the ERK1/2 pathway. *Innate Immunity* **26**, 505–513 (2020).

35.	Lu, J., Kang, J., Zhang, C. & Zhang, X. The role of IL-33/ST2L signals in the immune cells. *Immunology Letters* **164**, 11–17 (2015).

36.	Zhong, N. *et al.* High Mobility Group Box-1 Protein and Interleukin 33 Expression in Allergic Rhinitis. *Orl* **84**, 315–323 (2022).

37.	Arae, K. *et al.* Interleukin-33 and thymic stromal lymphopoietin, but not interleukin-25, are crucial for development of airway eosinophilia induced by chitin. *Sci Rep* **11**, 5913 (2021).

38.	Kawaguchi, K., Matsumoto, T. & Kumazawa, Y. Suppression of neuropeptide production by quercetin in allergic rhinitis model rats. *BMC Complementary and Alternative Medicine* **16**, 485 (2016).

39.	Li, H., Zhang, H. & Zhao, H. Apigenin attenuates inflammatory response in allergic rhinitis mice by inhibiting the TLR4/MyD88/NF-κB signaling pathway. *Environmental toxicology* **38**, 253–265 (2023).

40.	Li, H. *et al.* Enhancement of oral bioavailability of quercetin by metabolic inhibitory nanosuspensions compared to conventional nanosuspensions. *Drug Delivery* **28**, 1226–1236 (2021).

41.	Mu, D. *et al.* Quercetin-crosslinked chitosan nanoparticles: a potential treatment for allergic rhinitis. *Sci Rep* **14**, 4021 (2024).

42.	Naso M., Trincianti C., Tosca M. A. & Ciprandi G. Quercetin and Its Lecithin-Based Formulation: Potential Applications for Allergic Diseases Based on a Narrative Review. *Nutrients* **17**, 1476 (2025).

43.	Nakajima, S. *et al.* Resveratrol inhibits IL-33-mediated mast cell activation by targeting the MK2/3-PI3K/Akt axis. *Sci Rep* **9**, 18423 (2019).

44.	Sypka, M. *et al.* Mouse IgE clone SPE-7 can contain functional mouse IgG. *Allergy* **79**, 2544–2547 (2024).

45.	Comalada, M. *et al.* In vivo quercitrin anti-inflammatory effect involves release of quercetin, which inhibits inflammation through down-regulation of the NF-kappaB pathway. *Eur J Immunol* **35**, 584–592 (2005).

FIGURE LEGENDS

Fig. 1:**Quercetin exerts anti-inflammatory effects on IL-33-Induced RBL-2H3 cells.**

(a)Chemical structure of Quercetin. (b) Molecular docking models and predicted binding affinities (Vina scores) of Quercetin with IL-33 and ST2. (c) Cell viability assessed by CCK-8 assay after treatment with Quercetin (0.1-80 µM) for 24 or 48 hours. (d-e) Cytokine levels (IL-6, IL-13, TNF-α, MCP-1) in cell supernatant measured by ELISA. ST2-OE: ST2-overexpressing cells.Data are presented as mean ± SD (n = 3). Statistical comparisons were performed against the Control group (*), the IL-33-treated group (#), and the Vector-NC group (^). Significance levels: *, #, or ^ p < 0.05; **, ##, or ^^ p < 0.01; ***, ###, or ^^^ p < 0.001.

Fig. 2:**Quercetin attenuates IL-33-induced degranulation and apoptosis in activated RBL-2H3 cells via the IL-33/ST2/NF-κB signaling pathway.**

(a) Cell viability was measured by CCK-8 assay. (b) Histamine concentration and β-hexosaminidase release rate by ELISA. (c-d) Apoptosis by flow cytometry. The gating strategy is detailed in Supplementary Figure S1. (e-f) Protein levels of ST2, MyD88, p-IκBα/IκBα, NF-κB p65 by western blotting (β-actin as control). IgE group: Sensitized with anti-DNP-IgE and challenged with DNP-HSA. Data are presented as mean ± SD (n = 3). Lanes were cropped from different gels (each antibody was detected on a separate gel) and grouped for comparison; black lines indicate boundaries between gels. All samples were derived from the same experiment, and gels were processed in parallel. Full-length, uncropped blots with visible membrane edges are presented in Supplementary Figure 1**.** Statistical comparisons were performed against the Control group (*), the IgE group (#), IgE+IL-33-treated group (^). Significance levels: *, #, or ^ p < 0.05; **, ##, or ^^ p < 0.01; ***, ###, or ^^^ p < 0.001.

Fig. 3:**Quercetin reversed the enhanced degranulation and apoptosis induced by ST2 overexpression in RBL-2H3 cells.**

(a-b) ST2 protein levels by western blotting. (c) ST2 mRNA levels by qRT-PCR. (d) Cell viability by CCK-8 assay. (e) histamine concentration and β-Hexosaminidase release rate by ELISA. (f-g) Apoptosis by flow cytometry. (h) Protein levels of ST2, MyD88, IκBα, p-IκBα, NF-κB (p65) by western blotting (β-actin as control). Full-length, uncropped blots with visible membrane edges are presented in Supplementary Figure 1. Data are presented as mean ± SD (n = 3). Statistical significance: *p < 0.05, **p < 0.01, ***p < 0.001. among the compared groups.

Fig. 4: **Quercetin Attenuates Allergic Symptoms and Th2-Mediated Inflammation in OVA-Induced AR Mice.**

(a) Experimental timeline. (b, c) Sneezing and nasal rubbing counts (10 min, days 22–32). (d–g) Serum IL-4, IL-5, IL-13, OVA-sIgE levels. (h–i) IL-33 levels in serum and NLF. Data are presented as mean ± SD (n = 6). Statistical comparisons were performed against the Control group (*) and the Model group (#). Significance levels: * or # p < 0.05, ** or ## p < 0.01, *** or ### p < 0.001.

Fig. 5:**Quercetin restores histopathological changes in the nasal mucosa of OVA-induced AR mice.**

H&E and PAS staining of nasal mucosa from mice in each group (40× and 400× magnification, n = 3/group). Indicators: Red arrows = goblet cells; yellow arrows = epithelial thickness measurement; green arrows = inflammatory cell infiltration.)

Fig. 6:**Quercetin alleviates IL33/ST2/NF-κB pathway in OVA-induced AR mice.**

**(a)** Mucosal epithelial thickness. **(b)** Goblet cell density. **(c)** Representative western blot images of proteins in the IL-33/ST2/NF-κB pathway. **(d-f)** Quantitative analysis of protein levels of ST2, p-IκBα/IκBα, and NF-κB (p65), normalized to β-actin.
Data are presented as mean ± SD (n = 6 for A, B; n = 3 for D-F). Statistical comparisons were performed against the Control group (*) and the Model group (#). Significance levels: * or # p < 0.05, ** or ## p < 0.01, *** or ### p < 0.001.

Fig. 7:**Mechanism of Quercetin in Modulating Allergic Inflammation in OVA-Induced Allergic Rhinitis**

In the OVA-induced allergic rhinitis, type II inflammation is upregulated. Activated MCs release inflammatory cytokines and undergo degranulation, releasing histamine and triggering allergic inflammatory symptoms. Damaged epithelial cells release IL-33, which binds to the ST2 receptor on MCs. This interaction activates the NF-κB pathway, thereby amplifying the inflammatory response. Quercetin may exert anti-allergic inflammatory effects by modulating the IL-33/ST2/NF-κB signaling pathway.
